# Classical Swine Fever Outbreak after Modified Live LOM Strain Vaccination in Naive Pigs, South Korea

**DOI:** 10.3201/eid2404.171319

**Published:** 2018-04

**Authors:** Sang H. Je, Taeyong Kwon, Sung J. Yoo, Dong-Uk Lee, SeungYoon Lee, Juergen A. Richt, Young S. Lyoo

**Affiliations:** Konkuk University, Seoul, South Korea (S.H. Je, T. Kwon, S.J. Yoo, D.-U. Lee, Y.S. Lyoo);; HanByol Farm Tech, Namyangju, South Korea (S. Lee); Kansas State University, Manhattan, Kansas, USA (J.A. Richt)

**Keywords:** classical swine fever, modified live vaccine, residual virulence, pigs, South Korea, outbreak, vaccination, livestock, Jeju Island, LOM strain, viruses, classical swine fever virus

## Abstract

We report classical swine fever outbreaks occurring in naive pig herds on Jeju Island, South Korea, after the introduction of the LOM vaccine strain. Two isolates from sick pigs had >99% identity with the vaccine stain. LOM strain does not appear safe; its use in the vaccine should be reconsidered.

Classical swine fever is a highly contagious disease of pigs that tremendously affects the swine industry. Although several countries have become free from classical swine fever after eradication programs, sporadic outbreaks continue to occur in most major pig-producing countries, and classical swine fever is endemic to some countries in Asia. Vaccination is regarded as one of the most effective tools to prevent and control classical swine fever. Modified live vaccines (MLVs) mainly containing C-strain have been used widely because of their safety and provide complete protection against virus challenge (*1*,*2*).

Since 1974, the LOM strain has been the MLV strain for classical swine fever in South Korea. As a result of the government’s classical swine fever eradication program, Jeju Island, South Korea, became a classical swine fever virus (CSFV)–free area, and vaccination efforts ceased there in 1999 (*3*). Strong prohibition of live pig trade has also contributed to the maintenance of CSFV-naive herds on Jeju Island for over a decade, although sporadic classical swine fever outbreaks have occurred in mainland South Korea, despite mandatory vaccination with the LOM strain (*4*). This study describes classical swine fever outbreaks in naive pig herds on Jeju Island caused by the MLV.

Since 2014, multiple classical swine fever outbreaks have occurred on Jeju Island (Technical Appendix Figure 1). Clinical manifestation is characterized by reproductive problems (including stillbirth and fetus mummification), lethargy, cutaneous hyperemia, and cyanosis of the ear in young pigs. Pathologic examination showed typical classical swine fever lesions (Figure). Clinical samples from 2 nonvaccinated herds in 2016 were submitted for laboratory analysis.

**Figure:**
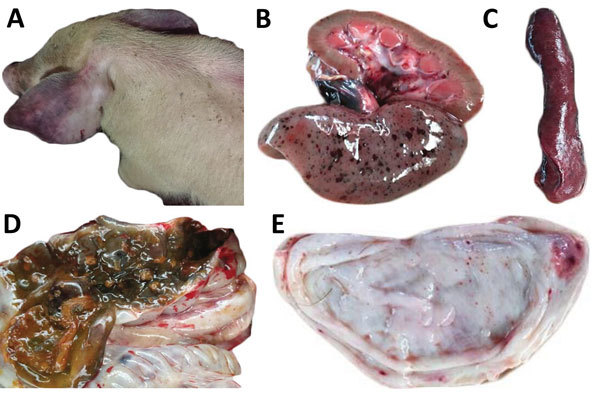
Clinical signs and pathologic lesions in naive pig infected with classical swine fever virus LOM vaccine strain, Jeju Island, South Korea. A) Cyanosis of ear. B) Hemorrhages in kidney. C) Marginal infarction of spleen. D) Button ulcers in large intestine. E) Hemorrhages in bladder.

PCR showed that these samples were positive for CSFV. Other viral pathogens involved in abortions (e.g., porcine reproductive and respiratory syndrome virus, Aujeszky disease virus, porcine parvovirus, Japanese encephalitis virus, and encephalomyocarditis virus) were not detected in any samples; however, lymph node, tonsil, lung, and brain fetal specimens and placenta specimens from farm A and lung specimens from farm B were weakly positive for porcine circovirus type 2, which is ubiquitous in South Korea (*5*). At farm B, serum samples from 20% of suckling piglets and 30% of weaned pigs were positive for CSFV. Although blood samples from growing and finishing pigs were not positive for CSFV, fecal samples were positive, indicating possible horizontal transmission in the field.

LOM isolates JJ-1601 (identified in a placenta sample from farm A) and JJ-1602 (in a spleen sample from farm B) shared 99.0% nucleotide identity with each other; and JJ-1601 shared 99.1% and JJ-1602 shared 99.5% nucleotide identity with the LOM strain. However, they shared low nucleotide identity (84%) with PC11WB, a virus isolated from a wild boar in South Korea (*6*). Phylogenetic analysis indicated that both viruses were classified within subgroup 1.1 (Technical Appendix Figure 2). Compared with LOM, JJ-1601 contained 5 aa and JJ-1602 10 aa substitutions in the N^pro^-E2 region; these substituions are not critical for acquisition of pathogenicity (Technical Appendix Table 2) (*7*).

In this study, we observed residual virulence of the LOM strain in naive herds. Since CSFV vaccine was accidentally introduced onto Jeju Island in 2014, continuous LOM outbreaks have occurred (Technical Appendix Table 3), resulting in tremendous damage to pig farms on the island. In addition, the virus has persistently circulated and caused repeated problems within the infected herds. Given that accidental vaccination was limited in 2014, the continuous classical swine fever outbreaks, including those occurring on farms A and B, resulted from farm-to-farm transmission of the vaccine virus strain.

CSFV live vaccination should guarantee safety to host animals: safety in young pigs, safety in pregnant sows, nontransmissibility, and no reversion to virulence (*8*). The first problem with the LOM vaccine was that the virus spread beyond initially introduced herds. Our results indirectly support horizontal transmissibility of the LOM vaccine within the infected herd. Another factor is the capacity of LOM to cause clinical signs in both young pigs and pregnant sows. Although we could not make observations in 2014 when the vaccine strain was first introduced, viruses with 99% nucleotide identity with LOM were found in CSFV-infected pigs that exhibited clinical signs and typical pathologic lesions of classical swine fever. This virulence could have occurred because of insufficient attenuation or reversion to virulence (*7*,*9*). In a previous study, vaccination of naive pregnant sows with LOM induced stillbirth and fetus mummification (*10*). These results suggested that transplacental transmission and fetal death might be inherent features of the vaccine, which indicates insufficient attenuation during virus adaption in vitro. Further study is needed to determine the basis for the virulence of the LOM strain in young pigs.

In conclusion, we must reconsider the use of LOM in the classical swine fever MLV and use a strain with experimental results satisfying safety requirements. Furthermore, control methods, including a marker vaccine for differentiating infected from vaccinated animals, are needed to stop the continuous damage and spread of LOM on Jeju Island.

Technical Appendix Methods for sequencing classical swine fever virus structural genes and phylogenetic analysis using these genes; methods for PCR and reverse transcription PCR for other porcine viruses; amino acid differences among LOM, JJ-1601, and JJ-1602; farms positive for LOM; and map of Jeju Island, South Korea.
