# Supplementary material for: Classical Swine Fever Outbreak after Modified Live LOM Strain Vaccination in Naive Pigs, South Korea
Source: Emerg Infect Dis. 2018 Apr;24(4):798–800. doi: 10.3201/eid2404.171319 (PMC5875281; doi:10.3201/eid2404.171319)
Supplement: Technical Appendix — Methods for sequencing classical swine fever virus structural genes and phylogenetic analysis using these genes; methods for PCR and reverse transcription PCR for other porcine viruses; amino acid differences among LOM, JJ-1601, and JJ-1602; farms positive for LOM; and map of Jeju Island, South Korea. [file 17-1319-Techapp-s1.pdf]

Article DOI: https://doi.org/10.3201/eid2404.171319

Classical Swine Fever Outbreak after Modified Live LOM Strain Vaccination in Naive Pigs, South Korea

Technical Appendix

Materials and Methods

A mummified fetus from farm A and a dead pig from farm B, both not vaccinated against classical swine fever virus (CSFV), were submitted (online Technical Appendix Figure 1). In addition, blood and fecal samples were also submitted from farm B for laboratory analysis. For farm A, we used spleen, lymph node, tonsil, lung, and brain samples from the mummified fetus and placenta for the detection of CSFV-specific RNA. For farm B, we used spleen, lymph node, tonsil, and lung tissue samples from a dead pig. We suspended tissue and fecal samples in Dulbecco's modified Eagle medium and homogenized. These homogenized samples, along with blood samples, were centrifuged at 3,000 rpm for 10 min. We processed the supernatant and serum fractions further.

Total RNA was extracted from 200 µL of serum or supernatant using Qiazol Lysis Reagent (QIAGEN, Germantown, MD, USA) according to the manufacturer's instructions. With the extracted RNA, one-step reverse transcription PCR (RT-PCR) was performed using CSFV-specific primer sets with SuPrimeScript RT-PCR premix (GeNet Bio, Daejeon, South Korea) (online Technical Appendix Table 1). The structural genes of the CSFVs from both farms were amplified and sequenced using 2 sets of primers. We aligned the nucleotide sequences encoding the structural proteins with those of reference strains deposited in GenBank and constructed a phylogenetic tree using the maximum-likelihood method with 1,000 bootstrap replicates. The nucleotide sequences of the structural genes of the 2 vaccine-related strains were deposited in GenBank (accession nos. KX954607 and KX954608).

To determine if other pathogens besides CSFV that can also induce reproductive failures were present in the mummified fetus and dead pig, we performed PCR for porcine reproductive

and respiratory syndrome virus, porcine circovirus type 2, Aujeszky disease virus, porcine parvovirus, Japanese encephalitis virus, and encephalomyocarditis virus. In brief, viral DNA and RNA was extracted by using Viral Gene-Spin Kit (iNtRON Biotechnology Inc., Seongnam, South Korea) according to the manufacturer's instructions. To detect porcine reproductive and respiratory syndrome virus, we performed conventional RT-PCR using virus-specific primer sets and SuPrimeScript RT-PCR premix (GeNet Bio). To detect porcine circovirus type 2, we performed PCR assays using virus-specific primer sets and HS Prime Taq Premix (GeNet Bio) (online Technical Appendix Table 1). In addition, 2 commercially available kits were used to detect Aujeszky disease virus and porcine parvovirus (VDx Abortion MP PCR kit, MEDIAN Diagnostics Inc., Chuncheon, South Korea) and encephalomyocarditis virus and Japanese encephalitis virus (VDx Abortion MP RT-PCR II, MEDIAN Diagnostics Inc.).

Technical Appendix Table 1. Primer sequences used to detect viruses and sequence the structural genes of classical swine fever virus, Jeju Island, South Korea, 2016*

Purpose	Virus	Orientation	Sequence
Diagnosis	CSFV	Forward	5′-CTA GCC ATG CCC AYA GTA GG-3′
		Reverse	5′-CAG CTT CAR YGT TGA TTG T-3′
	PRRSV	Forward	5′-ATG GCC AGC CAG TCA ATC A-3′
		Reverse	5′-TCG CCC TAA TTG AAT AGG TG-3′
	PCV2	Forward	5′-CAC GGA TAT TGT AGT CCT GGT-3′
		Reverse	5′-CCG CAC CTT CGG ATA TAC TGT C-3′
Sequencing	CSFV-F1	Forward	5′-CTA GCC ATG CCC AYA GTA GG-3′
		Reverse	5′-CCG GGG TGC AGT TGT TWG T-3′
	CSFV-F2	Forward	5′-TGC CTA TGC CCT ATC ACC TTA-3′
		Reverse	5′-CTA ACA GTG CTA CTA CTA CCA AG-3′

*CSFV, classical swine fever virus; PCV2, porcine circovirus type 2; PRRSV, porcine reproductive and respiratory syndrome virus.

Technical Appendix Table 2. Differences in amino acids between LOM strain and 2 viruses identified in field samples, Jeju Island, South Korea, 2016

	Amino acid position												
Virus	57	143	311	352	386*	476	539	581	584	870*	871*	933	Accession no.
LOM	K	L	Y	Y	H	R	K	S	E	N	W	D	EU789580
JJ-1601	K	L	Y	Y	D	R	K	S	V	K	L	N	KX954607
JJ-1602	R	Q	H	H	D	S	R	P	E	K	L	D	KX954608

*****These mutations were identical to those of vaccine strains bottled in all 5 commercial vaccines (Y.S. Lyoo, unpub. data).

Technical Appendix Table 3. Farms with pigs infected with LOM strain, Jeju Island, South Korea, 2014–2017*

Farm type	2014	2015	2016	2017
Newly infected				
Accidental introduction	19			
LOM confirmed	1	16	20	18
Serology		6	6	
Repeated detection from previous year		3	13	NA
Total	20	25	39	18

*NA, not available.

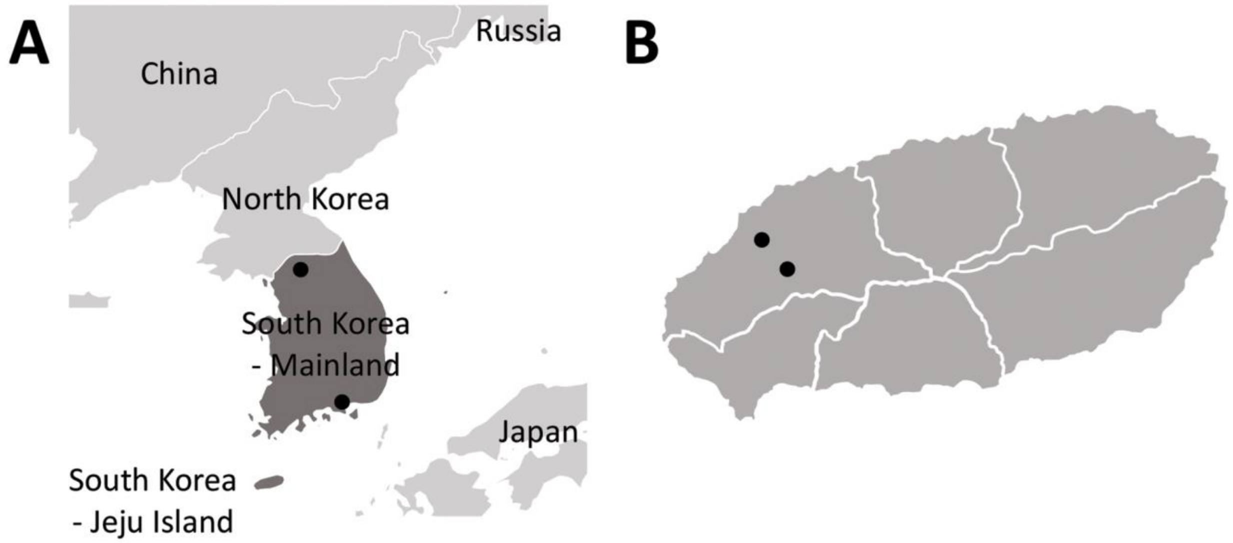

Technical Appendix Figure 1. Geographic location of mainland and Jeju Island of South Korea. A) Jeju Island is 80-km away from the South Korea mainland at the closest. Although 2 classical swine fever outbreaks caused by field strains occurred in domestic pig farms on the South Korea mainland, Jeju Island had maintained CSFV-free status without vaccination for over a decade. Lower and upper dots indicate classical swine fever outbreaks in 2014 and 2016. B) Geographic location of farms A and B (black dots; 5 km apart) on Jeju Island.

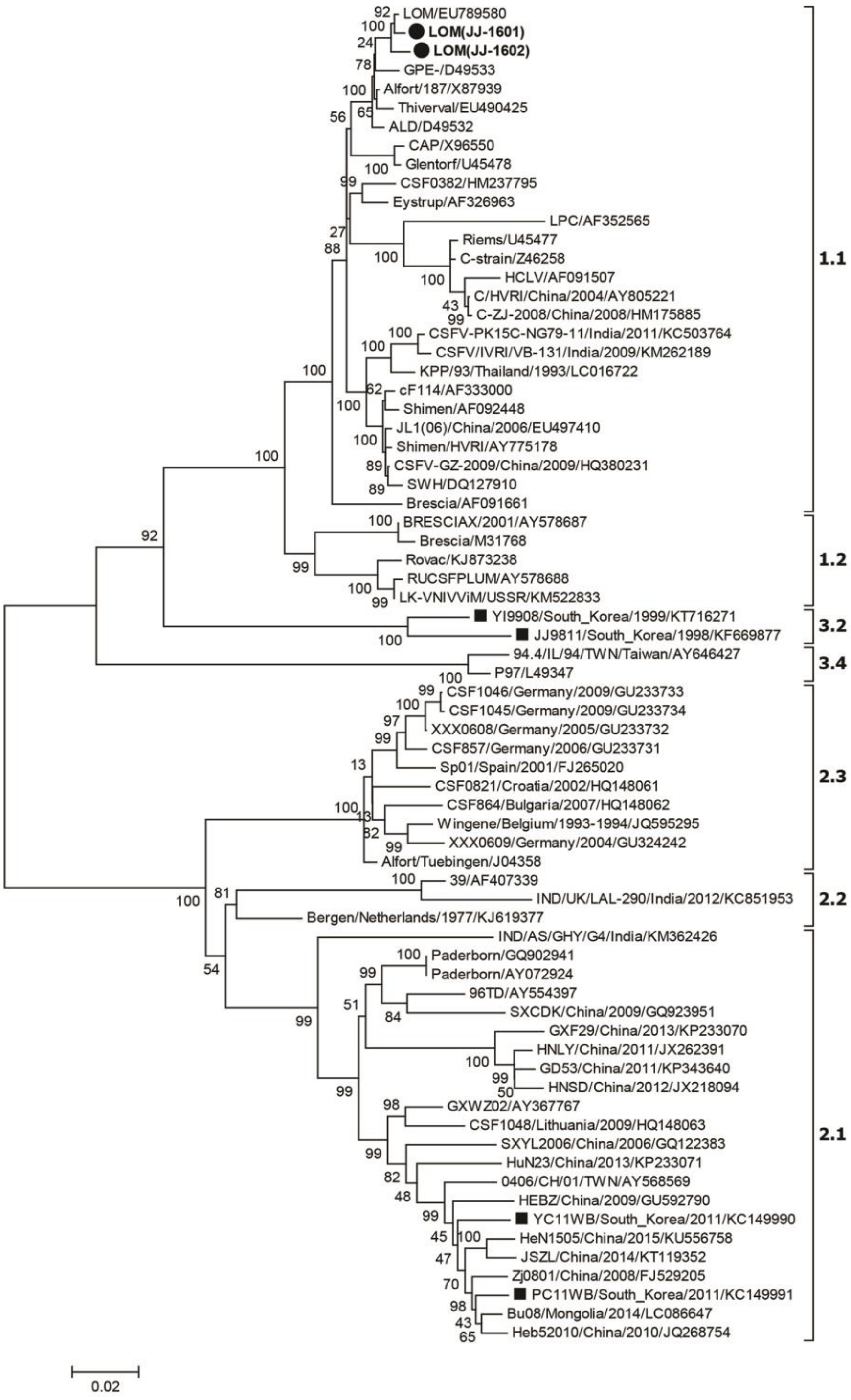

Technical Appendix Figure 2. Phylogenetic trees based on the whole structural genes of classical swine fever virus. The tree was constructed by using the maximum-likelihood method based on the general time-reversible plus gamma distribution plus invariable site model and tested by using 1,000 bootstrapping values. Black circles indicate the 2 isolates from a classical swine fever virus outbreak caused by the vaccine strain. Black squares indicate other field isolates found in South Korea.
